# Sarsasapogenin protects hair cells from cisplatin-induced ototoxicity by attenuating apoptosis and ferroptosis via alleviating oxidative stress

**DOI:** 10.3389/fphar.2025.1641174

**Published:** 2025-08-14

**Authors:** Zhifeng Chen, Ting Xie, Chenyu Chen, Tingting Lin, Xiaobo Wu, Yuqing Chen, Yanchun Lin, Xiaoyang Luo, Chaojun Zeng, Chang Lin

**Affiliations:** ^1^ Department of Otorhinolaryngology-Head and Neck Surgery, the First Affiliated Hospital, Fujian Medical University, Fuzhou, China; ^2^ Department of Otorhinolaryngology-Head and Neck Surgery, National Regional Medical Center, Binhai Campus of the First Affiliated Hospital, Fujian Medical University, Fuzhou, China; ^3^ Fujian Institute of Otolaryngology, the First Affiliated Hospital, Fujian Medical University, Fuzhou, China; ^4^ Department of Hepatopancreatobiliary Surgery, the First Affiliated Hospital, Fujian Medical University, Fuzhou, China; ^5^ ENT Institute and Department of Otorhinolaryngology, Eye & ENT Hospital, Fudan University, Shanghai, China; ^6^ NHC Key Laboratory of Hearing Medicine Research, Eye & ENT Hospital, Fudan University, Shanghai, China; ^7^ University of Science and Technology of China, Hefei, China; ^8^ Department of Otolaryngology, Affiliated Hospital of Putian University, Putian, China; ^9^ Putian Institute of Otolaryngology, Affiliated Hospital of Putian University, Putian, China

**Keywords:** sarsasapogenin, ototoxicity, cisplatin, apoptosis, ferroptosis, oxidative stress

## Abstract

**Background and Purpose:**

Cisplatin is a widely used chemotherapy drug for the treatment of solid tumours, but its clinical benefit is often limited by ototoxicity, leading to irreversible sensorineural hearing loss. However, there is a lack of effective strategies to prevent hearing loss caused by cisplatin in adults, while sodium thiosulfate is approved by the Food and Drug Administration in the United States for only use at the pediatric level. Sarsasapogenin, a natural compound of the *Anemarrhena asphodelides*, has antioxidant and neuroprotective properties, which suggest that it may attenuate the ototoxicity induced by cisplatin. The aim of this study is to evaluate the otoprotective effects of sarsasapogenin and its underlying mechanism as a potential therapeutic intervention for the prevention of ototoxicity induced by cisplatin.

**Methods:**

Cell viability was assessed by CCK-8 and cell apoptosis was assessed by flow cytometry. Reactive oxygen species (ROS) levels and mitochondrial dysfunction were quantified by immunofluorescence. In addition, expression of the molecules involved in apoptosis and ferroptosis was analyzed by qRT-PCR and Western blot. *In vivo* auditory function was evaluated by auditory brainstem response testing, and the survival of hair cells in the cochlea was quantified by immunolabeling with myosin-VIIa.

**Results:**

Sarsasapogenin significantly alleviated cisplatin-induced oxidative stress and restored mitochondrial function in House Ear Institute-Organ of Corti 1 (HEI-OC1) cells. Furthermore, sarsasapogenin effectively protected against cisplatin-induced sensorineural hearing loss and hair cell degeneration *in vivo*. Mechanistically, the protective effects of sarsasapogenin were primarily mediated through the inhibition of apoptosis and ferroptosis, both *in vitro* and *in vivo*.

**Conclusion:**

This study provides compelling evidence for the otoprotective effects of sarsasapogenin, suggesting its potential as a therapeutic intervention to prevent cisplatin-induced hearing loss.

## 1 Introduction

Hearing loss (HL) is a chronic disease prevalent worldwide and is increasingly recognized as an important public health problem today. The Global Burden of Disease Study (GBD) estimates that over 1.57 billion people were affected by hearing loss in 2019. By 2050, the number of individuals with hearing loss is expected to exceed 2.45 billion (GBD 2019 Hearing Loss Collaborators, 2021). There are various etiologies that contribute to HL, including cisplatin-induced hearing loss (CIHL). Cisplatin (Cis) is a platinum-based chemotherapy agent widely used to treat solid malignancies in adults and children, including head and neck, ovarian, cervical, breast, bladder, and lung cancers (Dasari and Tchounwou, 2014). Although cisplatin is clinically effective as an anticancer agent, its lack of selectivity between cancer and normal cells often results in significant side effects. Among these, ototoxicity stands out as a widespread and serious complication, primarily due to the accumulation and prolonged retention of the drug in the cochlear tissue (Breglio et al., 2017). Cisplatin-induced damage to hair cells (HCs), spiral ganglion neurons, and the stria vascularis results in bilateral, progressive, and irreversible sensorineural HL (Langer et al., 2013; Prayuenyong et al., 2021). CIHL is a common and serious adverse reaction affecting approximately 40%–60% of adult patients undergoing cisplatin therapy, with 18% experiencing severe to profound hearing loss following treatment (Frisina et al., 2016). In pediatric patients, the prevalence is even more concerning: up to 60% develop significant hearing loss after exposure to cisplatin (Knight et al., 2005). This hearing loss significantly impacts patients’ quality of life, poses significant challenges to their cognitive, psychological and social development, and affects their overall ability to communicate and learn effectively (Landier, 2016). Therefore, the development of effective treatment strategies to prevent and alleviate cisplatin-induced ototoxicity has become an urgent concern in clinical practice.

However, the mechanism underlying CIHL remains unclear. Oxidative stress plays a central role in the multifaceted process that leads to cisplatin-induced cochlear damage and subsequent hearing loss (Gentilin et al., 2019). Previous studies have shown that increased reactive oxygen species (ROS) production triggers mitochondria-mediated apoptosis in cochlear HCs (Steyger, 2021). The production of ROS and the degradation of antioxidant enzymes are the main molecular mechanisms underlying CIHL (Sheth et al., 2017). Oxidative stress leads to irreversible damage to cellular DNA, proteins, and lipids due to an imbalance between ROS production and antioxidant levels (Tan and Song, 2023), ultimately leading to lipid peroxidation and increased malondialdehyde and 4-hydroxynonenal levels (Rosati et al., 2019). Recent research has highlighted ferroptosis, a newly recognized non-apoptotic pathway of programmed cell death, as a contributing factor to cisplatin-induced ototoxicity. Ferroptosis is characterized by iron-dependent accumulation of lipid peroxides and a reduction in mitochondrial membrane potential and represents a distinct cellular process involved in the cochlear injury induced by cisplatin exposure (Mei et al., 2020). To prevent cisplatin-induced ototoxicity, multiple therapeutic strategies have been investigated in both clinical trials and preclinical models, including animal studies and cell lines, which primarily aim to prevent or mitigate hearing loss by targeting key mechanisms such as oxidative stress, apoptosis, and other cell death pathways. Antioxidant therapy has emerged as a key strategy for the treatment of CIHL (Tang et al., 2021). An increasing number of preclinical researches highlight the critical role of antioxidants in ameliorating the adverse hearing effects associated with cisplatin treatment. However, to date, no pharmacological intervention has been identified that can completely alleviate cisplatin-induced ototoxicity without compromising the efficacy of cisplatin or causing additional side effects (Yu et al., 2020). Therefore, identifying novel and effective drugs to prevent CIHL is of great importance.

**FIGURE 1:**
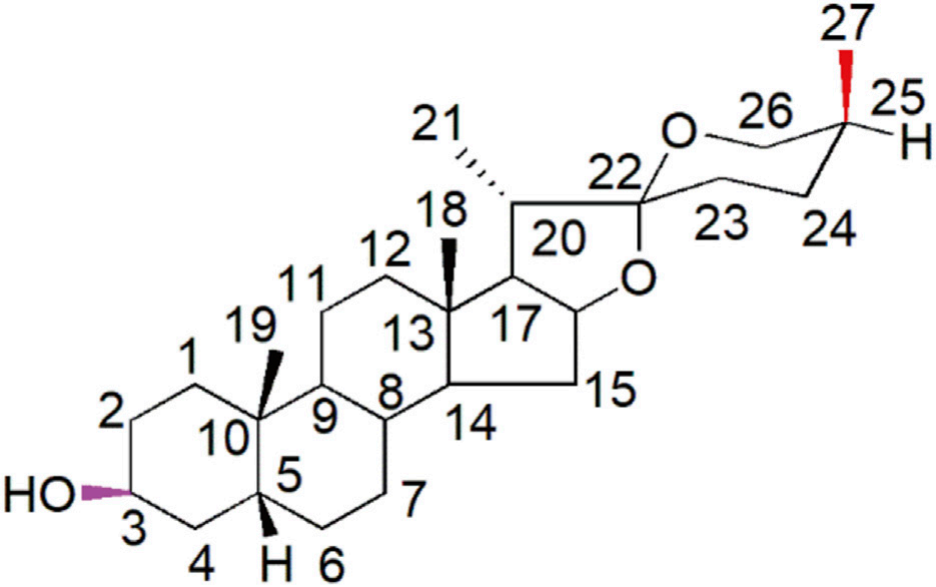
Structural representation of sarsasapogenin with the numerical designations assigned to each carbon atom explicitly indicated.

Sarsasapogenin (Sar) is a natural active ingredient classified as steroidal saponin isolated from Anemarrhena asphodeloides, a traditional Chinese medicinal plant. Sarsasapogenin is a spirostane glycoside sapogenin that has a 5β configuration between rings A and B (Figure 1) (Mustafa et al., 2022). Sarsasapogenin has been demonstrated to have potent antioxidant, anti-inflammatory, anti-osteoclastogenic and neuroprotective effects (Kashyap et al., 2020; Mandlik et al., 2021; Peng et al., 2020; Yu et al., 2021). Sarsasapogenin alleviated ulcerative colitis in rats by reducing proinflammatory mediators and oxidative stress (Mandlik et al., 2021). Furthermore, Sarsasapogenin inhibited lipopolysaccharide-induced inflammation and alleviated ear edema by reducing nitric oxide synthase expression and decreasing prostaglandin E2 levels in macrophages (Dong et al., 2017). The potent anti-inflammatory and antioxidant properties of sarsasapogenin suggest that it may provide a protective effect against cisplatin-induced ototoxicity. Despite these promising properties, the precise mechanisms by which sarsasapogenin can prevent or mitigate cisplatin-induced HCs damage remain to be fully elucidated.

In the present study, we used a combination of experimental models to examine cisplatin-induced damage, including the House Ear Institute-Organ of Corti 1 (HEI-OC1) cell line, which has similar properties to auditory hair cells. Furthermore, we used mouse cochlear explants and C57BL/6J mice to create comprehensive models of cisplatin-induced ototoxicity. These models were used to investigate the potential protective effect of sarsasapogenin against cisplatin-induced injury both *in vitro* and *in vivo*. Moreover, we aimed to elucidate the underlying mechanisms by which sarsasapogenin mitigates cisplatin-induced ototoxicity, thereby providing insights into its therapeutic potential in preventing hearing loss associated with cisplatin treatment.

## 2 Materials and methods

### 2.1 Animals

To avoid the effect of the estrous cycle in female mice on the experimental results, only male mice were used in this study (Shuster et al., 2021). Male C57BL/6J mice were purchased from the Shanghai Laboratory Animal Center and maintained in the laboratory animal room of the Laboratory Animal Center of Fujian Medical University. The experimental C57BL/6J mice were maintained in a temperature-controlled environment with a 12-h light/dark cycle to ensure a consistent circadian rhythm. They had unrestricted access to food and water throughout the study. All experiments were performed in strict accordance with the ARRIVE and “3Rs” guidelines (Percie du Sert et al., 2020). Every effort was made to minimize the use of animals. All experimental procedures were carried out in strict accordance with the ethical principles outlined in the Declaration of Helsinki and received formal approval from the Animal Ethics Committee of Fujian Medical University (IACUC FJMU 2022-0612).

### 2.2 Antibodies and reagents

The following antibodies and reagents were used: β-actin (Proteintech, 20536-1-AP, United States), CYCS (Proteintech, 10993-1-AP, United States), caspase 3 (Proteintech, 19677-1-AP, United States), GPX4 (Proteintech, 67763-1-Ig, United States), anti-myosin-VIIa rabbit antibody (*Proteus* Bioscience, 25–6790, United Kingdom), DAPI (Abcam, ab104139, United Kingdom), phalloidin (BioLegend, 424203, United States), cisplatin (MedChemExpress, HY-17394, United States), sarsasapogenin (MedChemExpress, HY-N0073, United States), and Liproxstatin-1 (MedChemExpress, HY-12726, United States).

### 2.3 Cell cultures

The HEI-OC1 cell line, a mouse auditory cell line, was kindly provided by Professor Wenyan Li from ENT Institute and Department of Otorhinolaryngology, Eye and ENT Hospital, Fudan University. Cells were cultured in high glucose Dulbecco’s Modified Eagle’s Medium (DMEM) supplemented with 10% fetal bovine serum (FBS; Gibco, A5670701, United States) to promote optimal cell growth and viability. To prevent bacterial contamination, 50 μg/mL ampicillin (Sangon Biotech, A430258-0200, China) was added to the medium. Culture conditions were adjusted to 33°C in a 10% CO2 environment to promote optimal growth. Once cells reached 80% confluence, they were sub-cultured with 0.25% trypsin/EDTA solution (Gibco, 25300054, United States) to ensure uniform passage and maintenance.

### 2.4 Organotypic culture of cochlear explants

Cochleae were harvested from P3 C57BL/6J mice and grown in DMEM/F12 growth medium (Gibco, 11330032, United States) supplemented with B-27 supplement (Gibco, 17504044, United States), N-2 supplement (Gibco, 17502001, United States), and 50 μg/mL ampicillin (Sangon Biotech, A5354-10ML, China). Cultures were maintained in a humidified incubator with 5% CO2 at 37°C. After overnight incubation, the cochlear explants in the experimental groups were exposed to cisplatin with or without sarsasapogenin, for 24 h. After the treatment period, the conditioned medium was removed and the explants were then incubated in fresh growth medium for an additional 48 h to allow recovery.

### 2.5 Cochlear HCs counts

To quantify surviving HCs in cochlear, myosin-VIIa immunolabeling was used. After treatment of the cochleae with sarsasapogenin, cisplatin, or a combination of both, immunestained hair cells were counted along 100-μm segments in each of the three cochlear turns (apical, middle, and basal). All measurements were performed in triplicate to ensure consistency and reliability of results.

### 2.6 Cell viability

HEI-OC1 cells were harvested by trypsinization with 0.25% trypsin/EDTA followed by centrifugation at 1,000 rpm for 3 min. The collected cells were resuspended in fresh culture medium and seeded in 96-well plates at a density of 4,000 cells per well, with three replicates for each condition. After a 24-h incubation period, the culture medium was replaced with medium containing different concentrations of cisplatin (0, 5, 10, 20, 30, and 40 μM) or sarsasapogenin (0, 10, 20, 30, 40, and 50 μM) and 20 μM cisplatin along with different concentrations of sarsasapogenin (0, 10, 20, 30, 40, and 50 μM). Cell viability was assessed at different incubation times (0.5–2 h) using the CCK-8 assay kit (NCM Biotech, C6005, China) to evaluate the cytoprotective effects of sarsasapogenin under cisplatin-induced stress.

### 2.7 Immunofluorescence analysis

Total ROS and mitochondrial ROS (mtROS) levels were measured using DCFH-DA (Beyotime, SOO33S, China) and MitoSOX Red (Thermo Fisher, M36008, United States), respectively. Mitochondrial membrane potential (MMP) was assessed using Tetramethylrhodamine ethyl ester perchlorate (TMRE, Beyotime, C2001S, China) according to the manufacturer’s instructions. FerroOrange staining was performed according to the manufacturer’s protocol using the FerroOrange kit (Dojindo, F374-10, Japan). First, samples were fixed in 4% paraformaldehyde (Sangon Biotech, Shanghai) for 1 h at room temperature to preserve cell integrity, followed by three washes with 0.01 M phosphate-buffered saline (PBS) (Sangon Biotech, Shanghai) to remove residues to remove fixative. Subsequently, permeabilization with 1% Triton X-100 (Solarbio Life Sciences, T8200, China) was performed for 30 min at room temperature to facilitate antibody access to the cells. To block nonspecific binding, samples were incubated with 5% bovine serum albumin (BSA; Solarbio Life Sciences, A8010, China) for 1 h. After blocking, samples were incubated with primary antibodies diluted 1:100 or 1:500 for 12 h at 4°C. After incubation with primary antibodies, samples were washed three times with PBS and incubated with secondary antibodies (ImmunoWay, RS3211, RS3611, RS3208 and RS3608, United States) for 1 h at room temperature. Finally, samples were washed three times after secondary antibody incubation. Cells were stained with DAPI (Abcam, ab104139, United Kingdom) to visualize nuclei, mounted on slides and prepared for imaging. Confocal microscopy was performed using an LSM 800 confocal microscope (Zeiss, Germany) to capture high-resolution images. All images were processed and analysed using the ImageJ software.

### 2.8 Flow cytometry

Cell apoptosis was assessed using an Annexin V-FITC/PI apoptosis detection kit (Yeasen, 40302ES50, China). HEI-OC1 cells cultured under different experimental conditions were harvested by trypsinization, followed by centrifugation at 1,500 rpm for 5 min to pellet the cells. The resulting cell pellets were washed twice with PBS to remove any residual culture medium or cell debris. After washing, cells were resuspended in 1× binding buffer at a concentration of 1 × 10^6^ cells/mL to prepare for staining. To detect apoptosis and necrosis, 10 μL of propidium iodide (PI) and 5 μL of Annexin V-FITC were added to each sample. These dyes simultaneously stain necrotic (PI-positive) and apoptotic (Annexin V-FITC-positive) cells. To allow optimal binding of the reagents, the samples were incubated for 20 min in the dark at room temperature. After incubation, cells were immediately analyzed by flow cytometry to quantify the proportions of apoptotic and necrotic populations. To ensure the reliability and reproducibility of the results, all experiments were performed in triplicate.

### 2.9 Measurement of Fe^2+^

An iron content detection kit (Solarbio, BC4355, China) was used to measure Fe^2+^ levels in cochlear tissue according to the manufacturer’s protocol. The treated samples were lysed and centrifuged at 4,000 × g for 10 min at 4°C, and the supernatant was boiled with the detection reagent. After adding chloroform and centrifugation at 10,000 rpm, the absorbance at 520 nm was measured using a SpectraMax i3x spectrophotometer (Molecular Devices, United States). The protein concentration was determined using a BCA assay (Meilunbio, China) and the Fe^2+^ values were normalized to the protein content (µg/mg).

### 2.10 Cisplatin-induced hearing loss model

To investigate the *in vivo* protective effect of Sar against CIHL, age-matched wild-type (WT) C57BL/6J mice aged 6–8 weeks with confirmed normal hearing were used. To induce hearing loss and damage to cochlear HCs, we prepared solutions containing 1 mg/mL cisplatin and a combination of 1 mg/mL cisplatin and 20 mM sarsasapogenin. Cisplatin was administered to mice via transtympanic injection according to established protocols for inducing hearing loss (Jiang et al., 2023). Mice with normal basic hearing were treated by injecting 0.01 mL of a warmed solution containing both cisplatin and sarsasapogenin into the left ear, while the right ear received an identical amount of cisplatin alone and served as a control. Auditory brainstem responses (ABRs) were recorded bilaterally before injection and again 3 days after injection to assess effects on auditory function.

### 2.11 Electrophysiological evaluation

Before injection, ABRs were recorded to confirm the normal hearing function of the mice. Throughout the procedure, animals were kept under general anesthesia induced with 1% sodium pentobarbital (75 mg/kg), and their body temperature was carefully maintained at 37°C using a thermostatically controlled heating pad. The ABR equipment (Neuro-Audio, Russia) has been strictly calibrated to ensure its accuracy and reliability. This process was carried out by the National Institute of Metrology of China (Report No. LSsx 2022-00028) according to standardized procedures to ensure precision in hearing measurements and to verify that the equipment meets all necessary technical specifications for use in the experimental protocols. The acoustic stimuli were delivered to a single ear via a plastic tubing system configured in a closed field setup. Subcutaneous electrodes were strategically placed for accurate signal detection, with the active electrode at the vertex of the head, the reference electrode under the mastoid of the ear to be tested, and the ground electrode at the mastoid of the ear on the other side. The acoustic stimulus was performed over a frequency range of 8–30 kHz, initially presented with a maximum sound pressure level (SPL) of 90 dB and gradually reduced in 5 dB steps until a sound pressure level of 0 dB was reached. The ABR threshold was defined as the lowest sound pressure level at which a clear wave I response was no longer evident.

### 2.12 Real-time quantitative PCR (RT-qPCR)

Total RNA was isolated from cochlear tissues and HEI-OC1 cells using FastPure Cell/Tissue Total RNA Isolation Kit (Vazyme, RC101-01, China) according to the manufacturer’s protocol. The extracted RNA was reverse transcribed into complementary DNA (cDNA) using RNA to cDNA EcoDry Premix (Takara, 639548, Japan). Subsequently, qPCR was performed using TB Green^®^ Premix Ex Taq™ (Takara, RR420A, Japan). All experimental procedures were performed according to the manufacturer’s instructions. The primer sequences used for qPCR are listed in Table 1. Actin served as an internal reference gene for normalization. Relative gene expression was determined using the comparative cycle threshold (ΔΔCt) method, which allowed quantification of gene expression changes between experimental groups.

**TABLE 1:** The primer sequences used for RT-qPCR.

Gene	Primer sequence
Mouse *β*-Actin forward	TGT​CCA​CCT​TCC​AGC​AGA​TGT
Mouse *β*-Actin reverse	AGC​TCA​GTA​ACA​GTC​CGC​CTA​G
Mouse *Bax* forward	AGA​TGA​ACT​GGA​CAG​CAA​TAT​GG
Mouse *Bax* reverse	GAT​CAG​CTC​GGG​CAC​TTT​AG
Mouse *Bcl2* forward	AGC​CTG​AGA​GCA​ACC​CAA​TG
Mouse *Bcl2* reverse	GAC​GGT​AGC​GAC​GAG​AGA​AG
Mouse *Casp3* forward	ATG​GGA​GCA​AGT​CAG​TGG​AC
Mouse *Casp3* reverse	GTC​CAC​ATC​CGT​ACC​AGA​GC
Mouse *CYCS* forward	TGG​ACC​AAA​TCT​CCA​CGG​TC
Mouse *CYCS* reverse	GGG​TAT​CCT​CTC​CCC​AGG​TG
Mouse *PARP* forward	CTT​GGT​GGA​GTA​CGA​GAT​TGA​C
Mouse *PARP* reverse	GAG​TGT​AGA​AGC​GAT​TGG​AGA​G
Mouse *Gpx4* forward	AAT​CAA​GGA​GTT​TGC​AGC​CG
Mouse *Gpx4* reverse	CCA​CGC​AGC​CGT​TCT​TAT​CA

### 2.13 Western blot (WB)

Cochlear tissue and HEI-OC1 cells were lysed using RIPA buffer (Meilunbio, China) supplemented with protease inhibitor cocktail (NCM Biotech, P001, China) to prevent protein degradation. Samples were incubated for 30 min at 4°C to ensure complete cell lysis. Protein concentrations were quantified using the BCA Protein Assay Kit (Meilunbio, China), and all samples were adjusted to equal protein concentrations and volumes. Equal amounts of protein were then loaded onto 10% or 12.5% sodium dodecyl sulfate-polyacrylamide gels (SDS-PAGE) for electrophoretic separation. After electrophoresis, the proteins were transferred to nitrocellulose membranes. To block nonspecific binding sites, the membranes were incubated with 5% nonfat dry milk in TBST buffer (Sangon Biotech, China) for 1 h at room temperature. Immunoblotting was performed using primary antibodies as described in the “Materials and Reagents” section. After overnight incubation with the primary antibodies at 4°C, the membranes were washed three times with TBST buffer and then incubated with appropriate secondary antibodies (Proteintech, SA00001-2, China) for 2 h at room temperature. Protein bands were visualized by treating the membranes with an enhanced chemiluminescence (ECL) detection kit (Meilunbio, MA0186, China) and imaging with a ChemiDoc XRS imaging system (Bio-Rad, United States). The intensity of protein bands was quantified using ImageJ software (NIH, Bethesda, MD, United States). To ensure reproducibility and statistical reliability, all experiments were performed in triplicate.

### 2.14 Statistical analysis

All data are presented as mean ± standard deviation (SD) and each experiment was performed at least three times to ensure consistency and reproducibility of results. Statistical analyzes were performed using GraphPad Prism (version 8.0.2; San Diego, CA, United States). For comparisons between two groups, an independent t-test was used, while for comparisons with more than two groups, one-way analysis of variance (ANOVA) followed by Dunnett’s *post hoc* test was used. A *p* value of <0.05 was considered statistically significant. In the organ explant culture experiments, “n” denotes the number of independent cochlear samples, whereas in the HEI-OC1 cell culture experiments, “n” represents the number of independent cell culture replicates.

## 3 Results

### 3.1 Sarsasapogenin protects HEI-OC1 cells from cisplatin-induced ototoxicity

**FIGURE 2:**
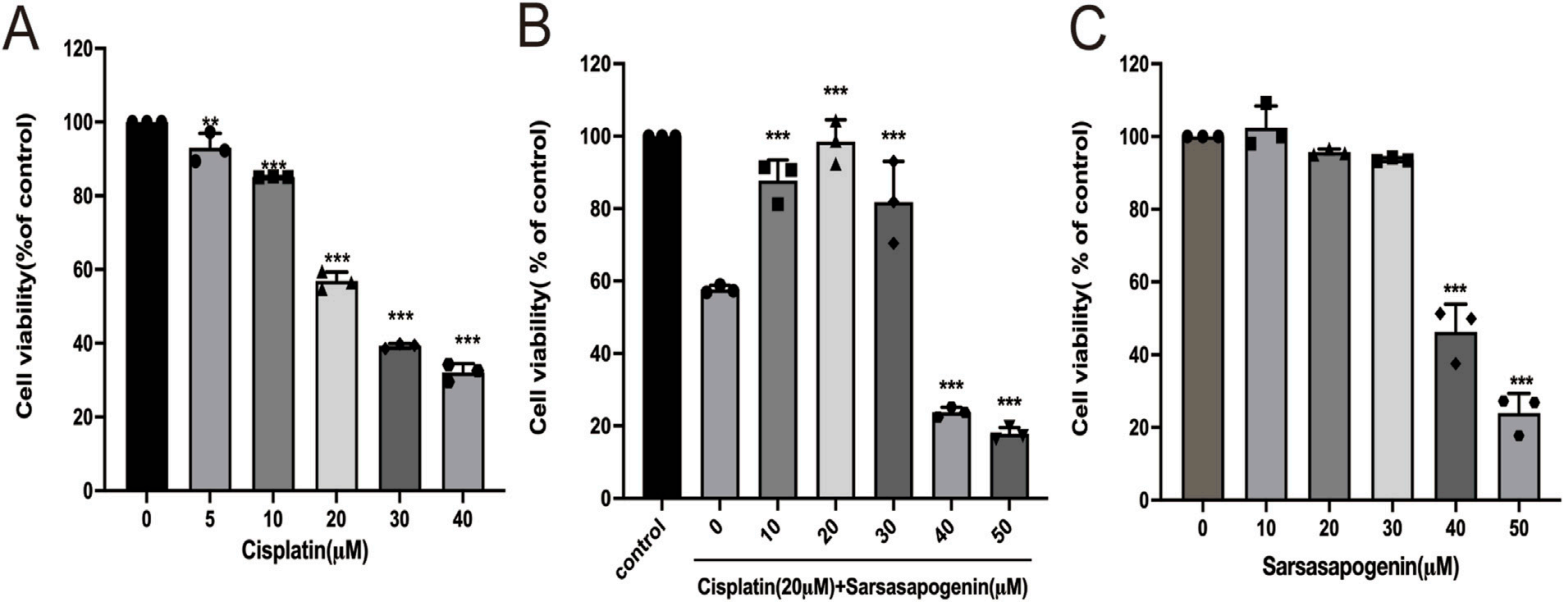
Sarsasapogenin protects HEI-OC1 cells from cisplatin-induced damage. **(A)** CCK-8 assay showing the viability of HEI-OC1 cells after treatment with different doses of cisplatin (0, 5, 10, 20, 30, and 40 µM) (n = 3). **(B)** CCK-8 assay showing the viability of HEI-OC1 cells after treatment with cisplatin (Cis; 20 µM) and various doses of sarsasapogenin (Sar; 0, 10, 20, 30, 40, and 50 µM) (n = 3). **(C)** CCK-8 assay showing the viability of HEI-OC1 cells after treatment with sarsasapogenin (0, 10, 20, 30, 40, and 50 µM) (n = 3). ^**^ *p* < 0.01, ^***^ *p* < 0.001.

To establish an optimal *in vitro* model for cisplatin-induced ototoxicity, HEI-OC1 cells were treated with increasing concentrations of cisplatin (0, 5, 10, 20, 30, and 40 μM) for 24 h, and to test cell viability assessed using the CCK-8 assay. A dose-dependent reduction in cell viability was observed compared to the untreated control group (Figure 2A). Notably, treatment of HEI-OC1 cells with 20 μM cisplatin for 24 h resulted in a significant reduction in cell viability (56.88% ± 2.41%). In contrast, treatment of HEI-OC1 cells with 30 μM cisplatin for 24 h resulted in cell viability below 50% (39.26% ± 0.65%). Based on these results, 20 μM cisplatin was selected for further *in vitro* experiments. To evaluate the protective effect of sarsasapogenin on HEI-OC1 cells after cisplatin exposure, HEI-OC1 cells were pretreated with different concentrations of sarsasapogenin (0, 10, 20, 30, 40, and 50 μM) for 4 h, followed by simultaneous treatment with 20 μM cisplatin for an additional 24 h. A significant cytoprotection effect was observed at sarsasapogenin concentrations of 10 and 20 μM, with the optimal protective effect achieved at 20 μM (Figure 2B). Furthermore, sarsasapogenin at concentrations of 10, 20, and 30 μM did not damage the HEI-OC1 cells. However, cell viability decreased below 50% at concentrations of 40 and 50 μM (Figure 2C). These results suggested that sarsasapogenin significantly increased the viability of HEI-OC1 cells in the presence of cisplatin-induced ototoxicity *in vitro*.

### 3.2 Sarsasapogenin attenuates cisplatin-induced oxidative stress and mitochondrial dysfunction in HEI-OC1 cells

**FIGURE 3:**
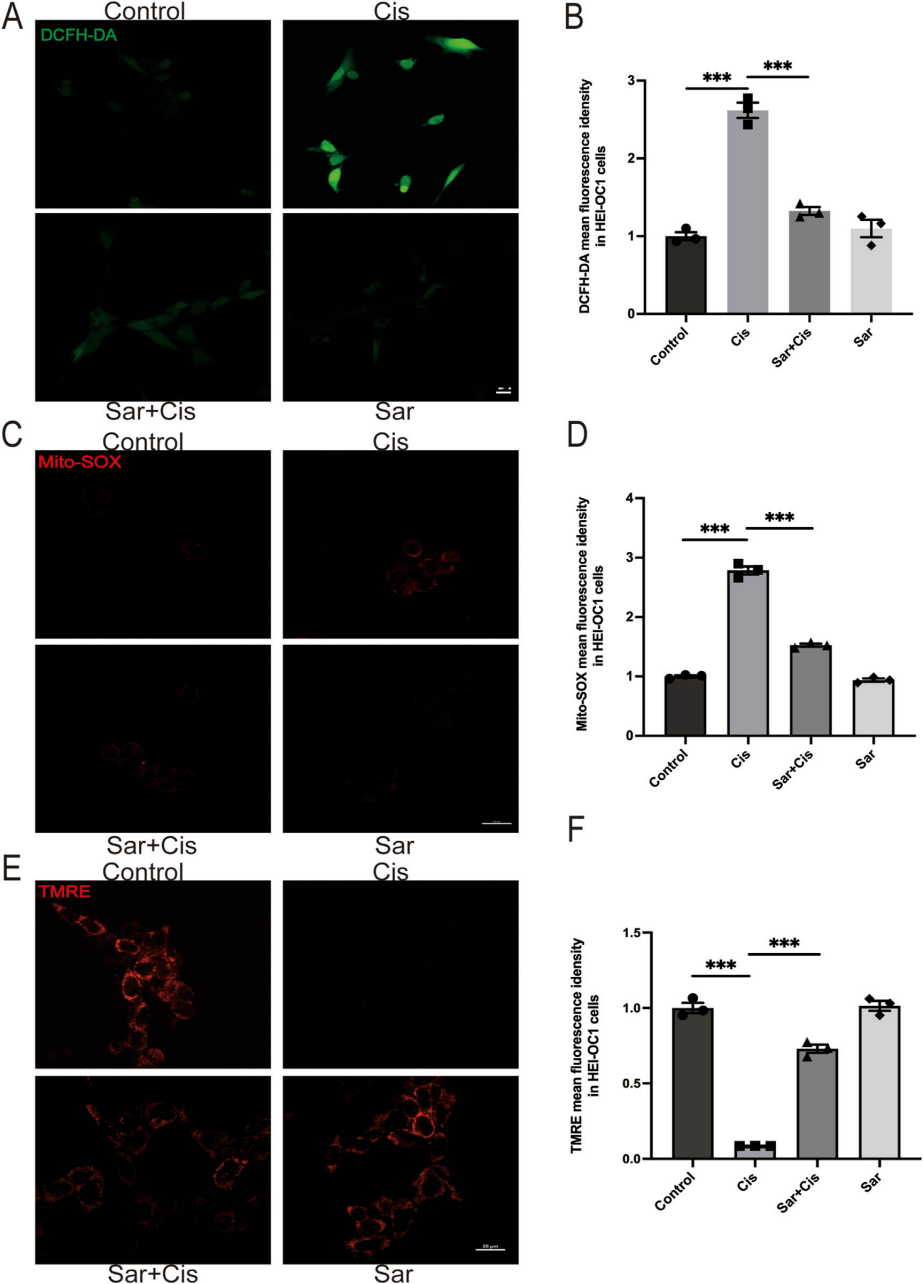
Sarsasapogenin inhibits cisplatin-induced oxidative stress and mitochondrial dysfunction in HEI-OC1 cells. **(A)** ROS production was increased by cisplatin and suppressed by cotreatment with sarsasapogenin (n = 3). **(B)** Quantitative analysis of DCFH-DA. **(C)** Mito-SOX staining showed that the increased mitochondrial ROS levels in HEI-OC1 cells after cisplatin treatment were alleviated by sarsasapogenin treatment (n = 3). **(D)** Quantitative analysis of Mito-SOX. **(E)** TMRE staining showed that the decreased MMP in HEI-OC1 cells after cisplatin treatment was restored by sarsasapogenin treatment (n = 3). **(F)** Quantitative analysis of TMRE. ^***^ *p* < 0.001. Scale bars: 20 µm.

Previous studies have shown that ROS generation is a key mediator of cochlear HCs damage associated with ototoxicity (Gentilin et al., 2019; Steyger, 2021). In the present study, we aimed to investigate whether sarsasapogenin provides a protective effect against cisplatin-induced HCs damage by inhibiting ROS production. To evaluate this hypothesis, we used two different fluorescent probes—DCFH-DA and MitoSOX Red—to measure total ROS and mtROS levels, respectively, in HEI-OC1 cells. A significant increase in both total ROS and mtROS levels after cisplatin exposure, whereas sarsasapogenin treatment markedly reduced ROS production (Figures 3A–D). Furthermore, previous studies have highlighted mitochondria as a major source of ROS, with ROS accumulation contributing to mitochondrial dysfunction in the cochlear (Fujimoto and Yamasoba, 2019). To further investigate whether sarsasapogenin can alleviate cisplatin-induced mitochondrial damage, we assessed MMP in HEI-OC1 cells using a combination of TMRE staining and immunofluorescence. Our results showed a significant reduction in TMRE intensity after cisplatin treatment, indicating mitochondrial dysfunction. In contrast, sarsasapogenin treatment significantly restored MMP (Figures 3E,F). Overall, these results suggested that sarsasapogenin protected against cisplatin-induced HC damage by attenuating oxidative stress and preventing mitochondrial dysfunction caused by ROS accumulation.

### 3.3 Sarsasapogenin protects HCs in mouse cochlear explants from *in vitro* cisplatin damage

**FIGURE 4:**
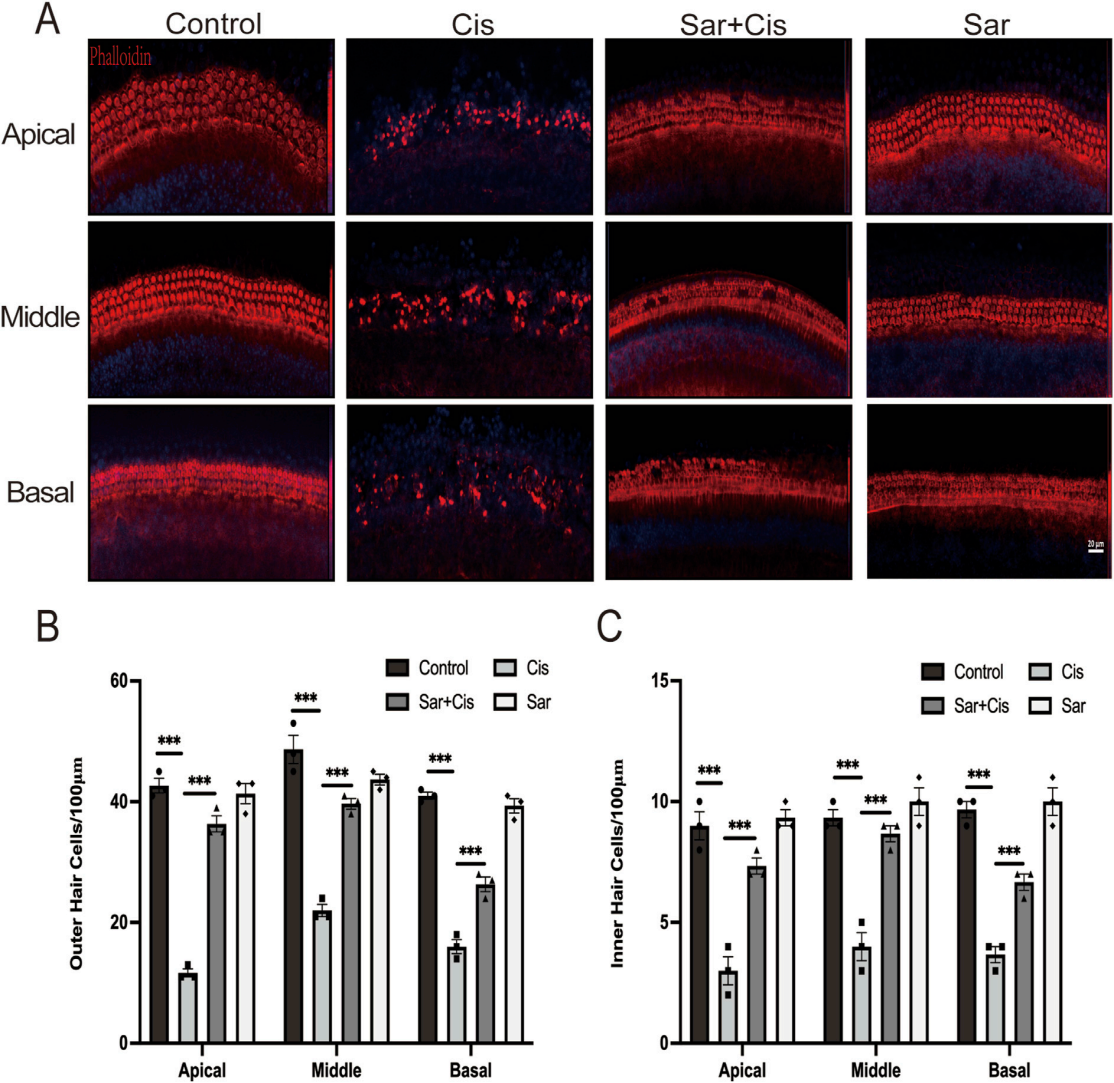
Effects of sarsasapogenin on cisplatin-induced damage to cochlear HCs. **(A)** Immunofluorescence staining for phalloidin and DAPI showing the number of HCs in different cochlear turns after different treatments (n = 3). **(B)** Quantitative analysis of phalloidin and DAPI-positive OHCs in **A**. **(C)** Quantitative analysis of phalloidin and DAPI-positive IHCs in **A**. ^***^ *p* < 0.001. Scale bars: 20 µm.

To assess whether sarsasapogenin can protect cochlear hair cells *in vitro*, cochlear explants from WT C57BL/6J mice from postnatal day 3 (P3) were used. The explants were assigned to four different experimental groups: the control group (no treatment), the cisplatin-only group (treated with 20 μM cisplatin for 24 h), the sarsasapogenin-only group (treated with 20 μM sarsasapogenin for 24 h), and the combined treatment group (pretreated with 20 μM sarsasapogenin for 4 h, followed by concurrent treatment with cisplatin and sarsasapogenin for 24 h. After treatment, cochlear explants were stained with DAPI (blue) and phalloidin (red) to specifically label HCs, which facilitated the quantification of HCs in the apical, middle, and basal regions of the cochlea in all experimental groups. As shown in Figure 4A, both the control group and the sarsasapogenin-only group showed no significant loss of HCs, suggesting that sarsasapogenin alone had no deleterious effects on the cochlear HCs. In contrast, the cisplatin-treated group showed a significant decrease in the number of double-positive cells (phalloidin and DAPI), indicative of HCs, within each cochlear turn (apical, middle, and basal) compared to the control group (Figures 4A–C). Notably, the cochlear explants in the cisplatin plus sarsasapogenin group showed significant preservation of HCs compared to the cisplatin-only group (Figures 4A–C). These results suggested that sarsasapogenin effectively attenuated cisplatin-induced HCs loss *in vitro*.

### 3.4 Sarsasapogenin protects cochlear HCs against CIHL in C57BL/6J mice *in vivo*

**FIGURE 5:**
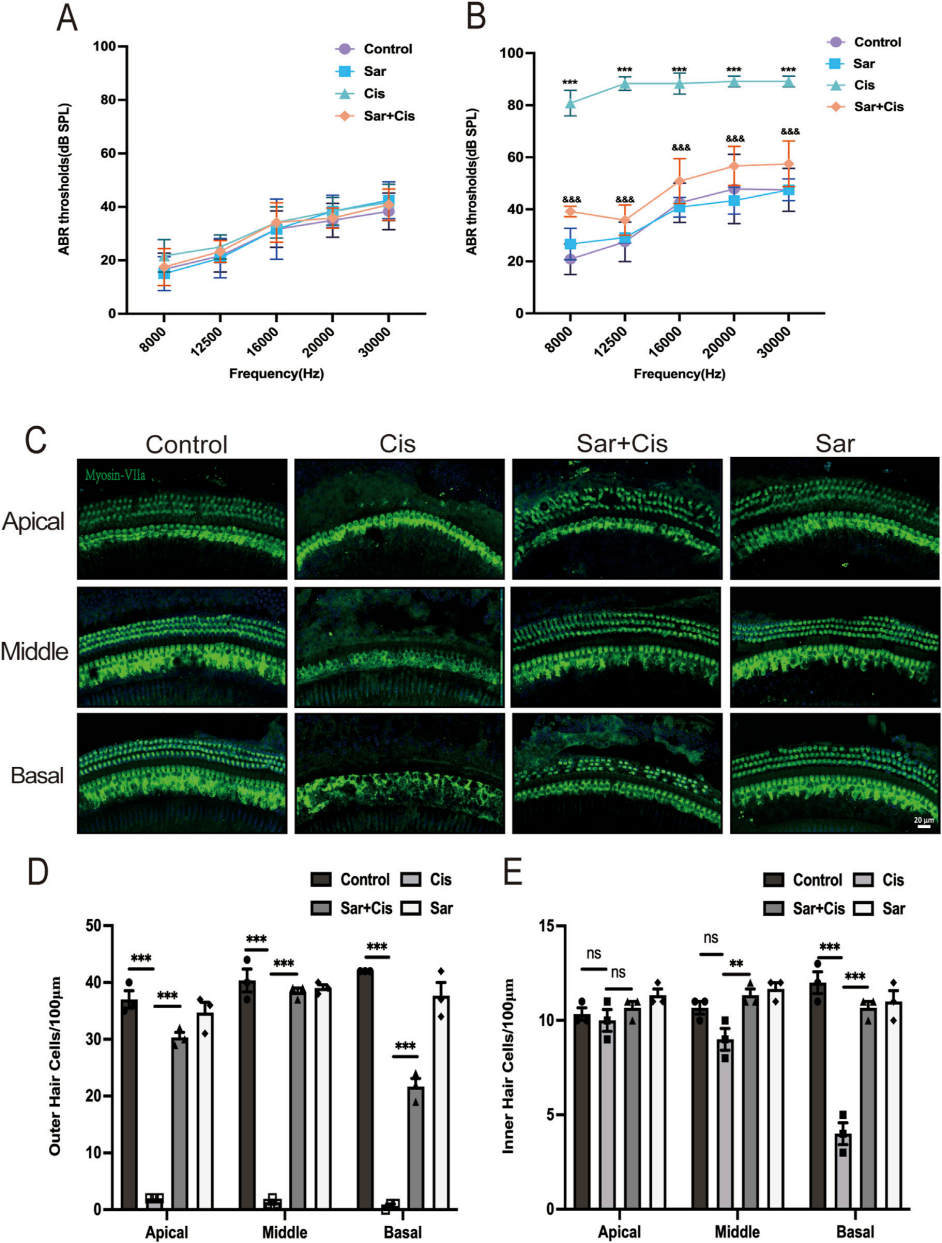
Sarsasapogenin ameliorates cisplatin-induced hearing loss and cochlear hair cell damage *in vivo*. **(A)** There was no difference in the ABR hearing threshold of C57BL/6J mice in the different groups before treatment (n = 6). **(B)** Transtympanic injection of sarsasapogenin significantly reduced the ABR threshold caused by cisplatin at 8, 12.5, 16, 20 and 30 kHz (n = 6). ^***^ *p* < 0.001 *versus* the control group; ^&&&^ *p* < 0.001 *versus* the cisplatin group. **(C)** Whole-mount sections were labeled with myosin-VIIa (green) and DAPI (blue). Sarsasapogenin treatment significantly improved HCs loss in all turns (n = 3). **(D)** Quantitative analysis of surviving OHCs from different turns. **(E)** Quantitative analysis of surviving IHCs from different turns. ns, not significant, ^**^ *p* < 0.01, ^***^ *p* < 0.001. Scale bars: 20 µm.

The experiments discussed above confirmed the protective effect of sarsasapogenin on both HEI-OC1 cells and cochlear explants. To further assess the efficacy of sarsasapogenin *in vivo*, we examined its effects on adult C57BL/6J mice. Due to the innate resistance of mice to cisplatin and the low survival rate after systemic cisplatin administration, a localized transtympanic injection approach was chosen for modeling purposes. Previous studies have demonstrated that transtympanic administration of cisplatin reliably produces ototoxicity in mice (Jiang et al., 2023). In this study, we also used a dose of 1 mg/kg cisplatin administered via transtympanic injection to establish a cisplatin-induced ototoxicity mouse model. Before treatment, an ABR test was performed to ensure normal hearing thresholds in all mice. Mice with normal hearing were divided into two groups: six mice received cisplatin only in the left ear (the Cis group) and a combination of sarsasapogenin and cisplatin in the right ear (the Sar + Cis group), while the remaining six mice received saline only in the left ear (the control group) and sarsasapogenin in the right ear alone, without cisplatin (the Sar group). ABR measurements were repeated 3 days after injection. Before modeling, no significant differences in hearing thresholds were observed in any group (Figure 5A). After treatment, the control and Sar groups showed no significant changes in hearing threshold, indicating that sarsasapogenin alone, administered at a certain concentration, did not affect hearing function. In contrast, the Cis group showed a significant increase in hearing threshold across all tested frequencies. However, in the Sar + Cis group, hearing thresholds at all frequencies were significantly improved compared to those in the Cis group (Figure 5B), suggesting a protective effect of sarsasapogenin against CIHL. Cochlear morphology was further analyzed by HC counting, yielding results consistent with the ABR data. In the Cis group, there was a significant decrease in cochlear HCs, with the greatest damage observed in the basal turn. In contrast, mice in the Sar + Cis group did not show significant HCs loss, demonstrating the robust protective effect of sarsasapogenin against cisplatin-induced cochlear damage (Figures 5C–E). These results highlighted the strong protective effect of sarsasapogenin in alleviating cisplatin-induced ototoxicity, as it not only preserved hearing function but also prevented the loss of cochlear HCs *in vivo*.

### 3.5 Sarsasapogenin suppresses cisplatin-induced apoptosis in HEI-OC1 cells and mouse cochlear explants *in vitro* and in mouse cochlear *in vivo*

**FIGURE 6:**
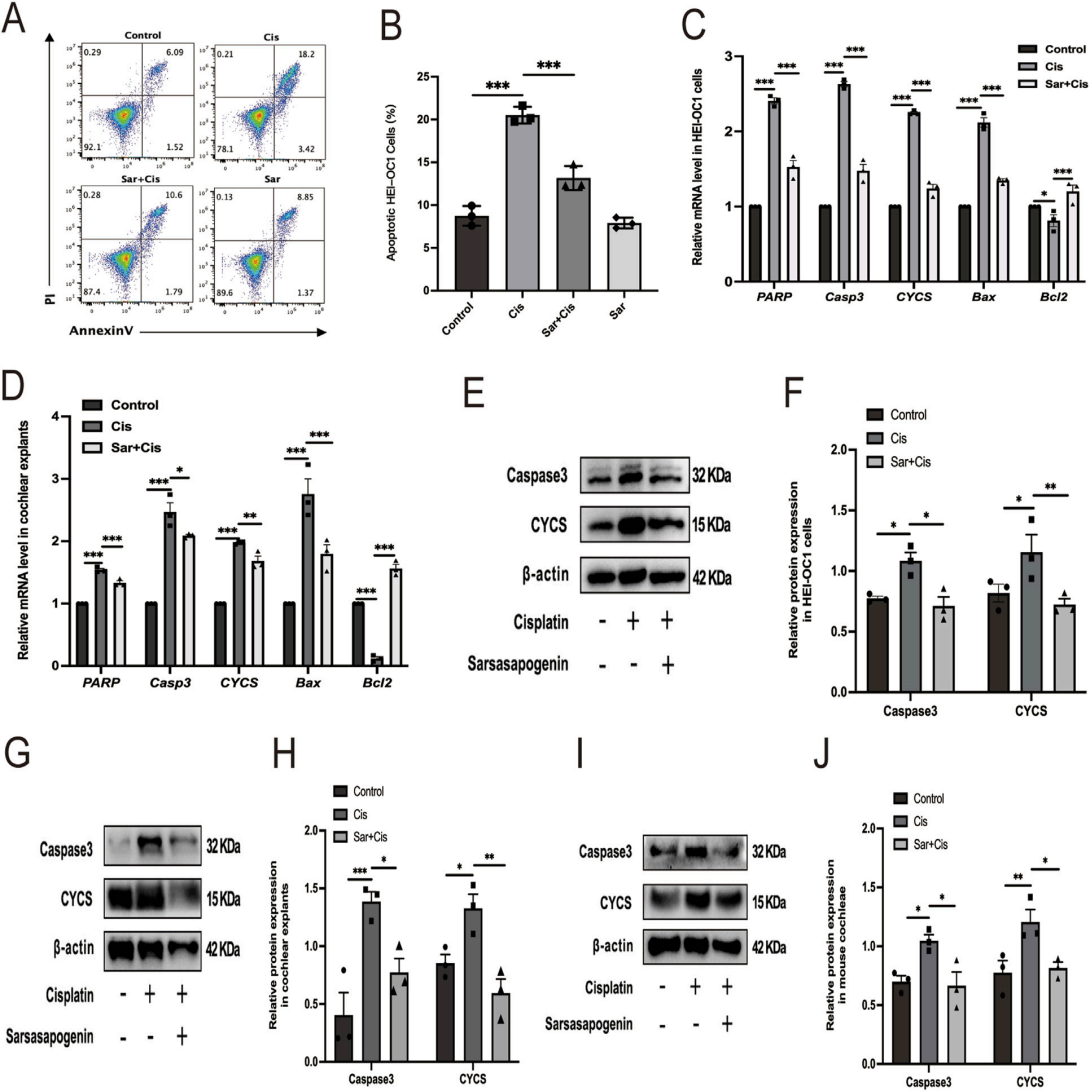
Sarsasapogenin suppresses cisplatin-induced apoptosis *in vitro* and *in vivo*. **(A)** Flow cytometry results showing the apoptosis rates in the different groups (n = 3). **(B)** Quantitative analysis of the flow cytometry results in **A**. **(C)** The mRNA expression profiles of apoptotic markers including *PARP*, *Casp3*, *CYCS*, *Bax*, and antiapoptotic marker *Bcl2* in HEI-OC1 cells after different treatments (n = 3). The values of qPCR were normalized to β-actin and averaged. **(D)** The mRNA expression of *PARP*, *Casp3*, *CYCS*, *Bax*, and *Bcl2* in cochlear explants after different treatments (n = 3). The values of qPCR were normalized to β-actin and averaged. **(E)** WB results showing the expression levels of caspase 3 (n = 3) and CYCS (n = 3) in the different groups in HEI-OC1 cells. **(F)** Quantitative analysis of the Western blot results in **E**. **(G)** WB results showing the expression levels of caspase 3 (n = 3) and CYCS (n = 3) in the different groups in cochlear explants. **(H)** Quantitative analysis of the Western blot results in **G**. **(I)** WB results showing the expression levels of caspase 3 (n = 3) and CYCS (n = 3) in the different groups in mouse cochleae. **(J)** Quantitative analysis of the Western blot results in **I**. ^*^ *p* < 0.05, ^**^ *p* < 0.01, ^***^ *p* < 0.001.

To comprehensively investigate whether the protective mechanism of sarsasapogenin is related to its ability to suppress cisplatin-induced apoptosis, we used an Annexin V-FITC/PI assay for cell labeling and flow cytometry for quantitative analysis and thus determined the precise percentage of apoptotic cells (Figure 6A). HEI-OC1 cells were divided into four different groups based on the treatment conditions: control, 20 μM cisplatin alone, 20 μM sarsasapogenin alone, and a combined treatment of 20 μM sarsasapogenin with 20 μM cisplatin. After 24 h of culture, sarsasapogenin alone had no significant effect on apoptosis, indicating minimal cytotoxicity. However, when cisplatin was introduced, co-treatment with sarsasapogenin significantly reduced the proportion of apoptotic cells compared to the group receiving cisplatin alone, suggesting that sarsasapogenin exerts a strong inhibitory effect on cisplatin-induced apoptosis. Quantitative analysis of flow cytometry data clearly demonstrated the protective effect of sarsasapogenin in the context of cisplatin toxicity (Figure 6B). This observation was further supported by RT-qPCR and WB analyzes. Quantitative analysis using RT-qPCR targeting both apoptosis-related genes, namely poly (ADP-ribose) polymerase (*PARP*), *Casp3*, cytochrome C (*CYCS*) and *Bax*, and the anti-apoptotic gene, namely *Bcl2*, targeted-further supported the anti-apoptotic effect of sarsasapogenin. Cisplatin exposure resulted in significant upregulation of proapoptotic markers, including *PARP*, *Casp3*, *CYCS*, and *Bax*, and significant downregulation of *Bcl2,* an antiapoptotic marker, indicating a strong apoptotic response (Figure 6C). However, in the sarsasapogenin-pretreated group, the expression levels of these apoptotic genes were significantly decreased and the expression of the anti-apoptotic gene was significantly increased compared to those in the group that received cisplatin alone. Similar results were observed in the cochlear explants (Figure 6D). The antiapoptotic ability of sarsasapogenin was further confirmed by the significant reduction in protein levels of caspase3 and CYCS in sarsasapogenin-pretreated groups compared to those treated with cisplatin alone *in vitro* and *in vivo* (Figures 6E–J). Overall, these results suggested that sarsasapogenin exerted a protective effect by significantly suppressing cisplatin-induced apoptosis both *in vitro* and *in vivo*.

### 3.6 Sarsasapogenin alleviates cisplatin-induced ferroptosis in HEI-OC1 cells and mouse cochlear explants *in vitro* and in mouse cochleae *in vivo*

**FIGURE 7:**
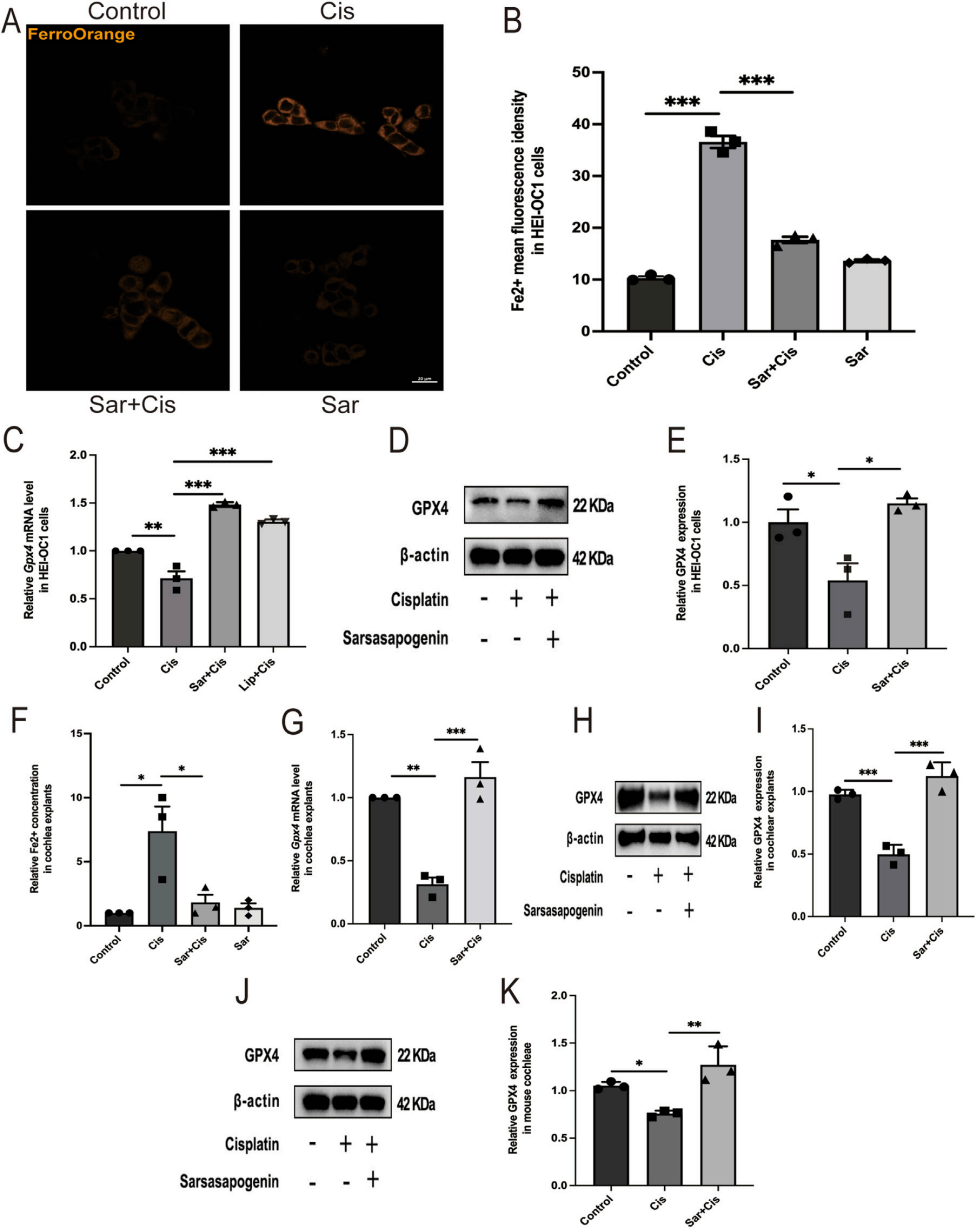
Sarsasapogenin alleviates cisplatin-induced ferroptosis *in vitro* and *in vivo*. **(A)** FerroOrange staining showed that the increased Fe^2+^ levels in HEI-OC1 cells after cisplatin treatment were alleviated by sarsasapogenin treatment (n = 3). **(B)** Quantitative analysis of FerroOrange staining. **(C)** The mRNA expression of the ferroptosis-related gene *Gpx4* in HEI-OC1 cells after different treatments (n = 3). The values of qPCR were normalized to β-actin and averaged. **(D)** WB results showing the expression levels of GPX4 (n = 3) in the different groups in HEI-OC1 cells. **(E)** Quantitative analysis of the Western blot results in **D**. **(F)** The significant increase in tissue-associated Fe^2+^ levels after cisplatin treatment in cochlear explants was alleviated by sarsasapogenin treatment (n = 3). **(G)** The mRNA expression of the ferroptosis-related gene *Gpx4* in cochlear explants after different treatments (n = 3). The values of qPCR were normalized to β-actin and averaged. **(H)** WB results showing the expression levels of GPX4 in the different groups in cochlear explants (n = 3). **(I)** Quantitative analysis of the Western blot results in **H**. **(J)** WB results showing the expression levels of GPX4 in the different groups in mouse cochleae (n = 3). **(K)** Quantitative analysis of the Western blot results in **J**. ^*^ *p* < 0.05, ^**^ *p* < 0.01, ^***^ *p* < 0.001. Scale bars: 20 µm.

To investigate the effect of sarsasapogenin on ferroptosis, we used the FerroOrange staining and an iron content assay kit to determine intracellular Fe^2+^ levels in HEI-OC1 and Fe^2+^ content in cochlear explants, respectively. The results showed a significant increase in Fe^2+^ levels after cisplatin treatment, whereas sarsasapogenin treatment significantly attenuated this increase both in HEI-OC1 cells and cochlear explants (Figures 7A,B,F). Additionally, we examined the expression of the ferroptosis-related gene glutathione peroxidase 4 (*Gpx4*). *Gpx4* expression was significantly higher in the sarsasapogenin-pretreated group than in the cisplatin-only group, both in HEI-OC1 cells and cochlear explants (Figures 7C,G). Furthermore, to better demonstrate that ferroptosis exists in cisplatin treated HEI-OC1 cells, 5 μm Liproxstatin-1 (Zheng et al., 2020) was applied to HEI-OC1 cells with cisplatin for 24 h. As expected, Liproxstatin-1 (Lip, ferroptosis inhibitor) reversed the expression of the ferroptosis-related gene *Gpx4* (Figure 7C). This observation was further confirmed by WB analysis, which showed that cisplatin significantly decreased GPX4 protein levels in HEI-OC1 cells and cochlear explants *in vitro* and cochlear tissues from C57BL/6J mice *in vivo*. However, pretreatment with sarsasapogenin significantly restored GPX4 protein levels *in vitro* and *in vivo* models (Figures 7D,E,H–K). Taken together, these results suggested that sarsasapogenin effectively alleviated cisplatin-induced ferroptosis both *in vitro* and *in vivo*.

### 3.7 Sarsasapogenin does not compromise the tumor-killing efficacy of cisplatin in cancer cell lines

**FIGURE 8:**
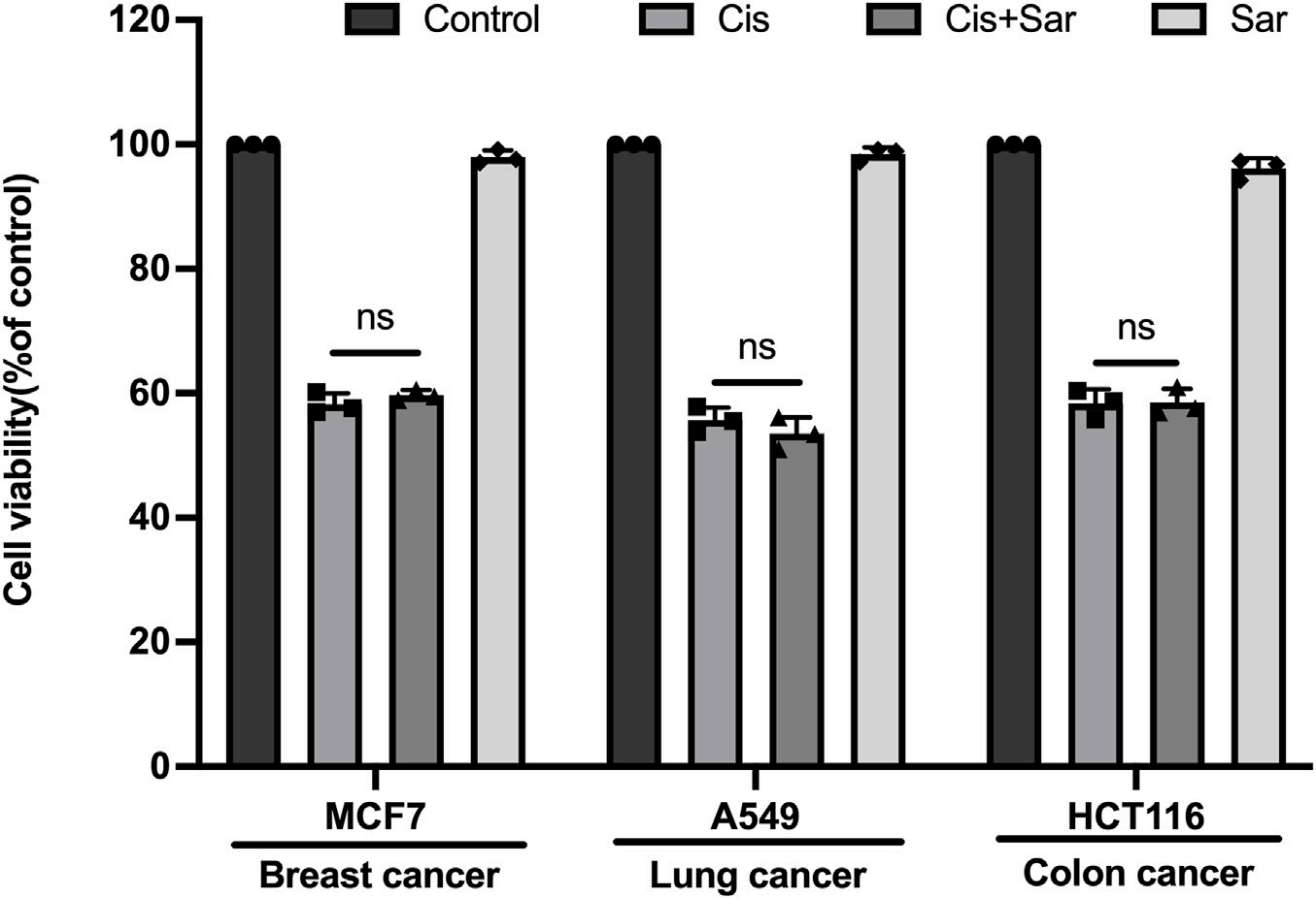
Sarsasapogenin has no effect on cisplatin’s ability to induce cancer cell death. CCK-8 assay showing the cell survival of several cancer cell lines treated with a combination of sarsasapogenin and cisplatin (n = 3). ns, not significant.

Although sarsasapogenin has been shown to protect against cisplatin-induced ototoxicity in various experimental models, it is important to evaluate whether sarsasapogenin affects the efficacy of cisplatin as a chemotherapeutic agent before considering its clinical use. To investigate this issue, we examined the influence of sarsasapogenin on the antitumor efficacy of cisplatin in three human tumor cell lines: MCF7 (breast cancer), A549 (lung cancer), and HCT116 (colon cancer). Each cell line was exposed to a 24-h treatment with a combination of sarsasapogenin and cisplatin. Cell viability was then determined using the CCK-8 assay. The experimental groups included cells treated with combination therapy of sarsasapogenin and cisplatin, while the control groups consisted of cells treated with medium alone, cisplatin alone, or sarsasapogenin alone. Viability was calculated as a percentage compared to the medium-only control (Figure 8). The results showed that sarsasapogenin did not affect the ability of cisplatin to trigger tumor cell death. Overall, these results suggested that sarsasapogenin did not interfere with the cytotoxic effects of cisplatin on tumor cells, suggesting that it can be used safely without affecting the chemotherapeutic efficacy of cisplatin.

## 4 Discussion

Cisplatin is widely used in clinical practice because of its effectiveness in the treatment of solid tumors and hematological malignancies (Wang and Lippard, 2005). Cisplatin exerts its antitumor effects through DNA alkylation, disrupting with DNA replication and repair mechanisms, ultimately leading to the death of proliferating tumor cells (Choi et al., 2015). However, cisplatin also produces non-specific toxicity in normal cells, leading to serious side effects such as neurotoxicity, hepatotoxicity, nephrotoxicity, and ototoxicity (Ghosh, 2019). The development of new therapeutics to ameliorate CIHL, which are progressive, irreversible, cumulative, and dose-dependent remains a critical area of research (Frisina et al., 2016). Sarsasapogenin, a naturally occurring compound, has attracted considerable attention due to its well-documented safety profile as well as its potent antioxidant, anti-inflammatory, and neuroprotective properties (Mandlik et al., 2021; Yu et al., 2021). In this study, we found that sarsasapogenin reduced cisplatin-induced HC damage by attenuating oxidative stress, rescuing mitochondrial dysfunction, and inhibiting apoptosis and ferroptosis, both *in vitro* and *in vivo*. Moreover, *in vivo* experiments demonstrated that transtympanic administration of sarsasapogenin effectively attenuated cisplatin-induced hearing loss and protected against the loss of HCs. In addition, sarsasapogenin did not affect the cytotoxic effects of cisplatin on tumor cells.

The present study demonstrated that sarsasapogenin provided significant protection against cisplatin-induced ototoxicity in both *in vitro* and *in vivo* models. We first examined its protective role *in vitro*, where sarsasapogenin significantly increased the viability of HEI-OC1 cells and increased the survival rate of HCs in cochlear explants. This suggests that sarsasapogenin is able to reduce cell damage caused by cisplatin exposure. We then extended our study by assessing ABR and performing quantitative analyzes of cochlear HCs using myosin-VIIa immunostaining. Consistent with previous studies, cisplatin treatment resulted in significant loss of OHCs in the basal, middle, and apical turns of the cochleae, accompanied by a significant increase in hearing threshold (Li et al., 2023; Lu et al., 2024). A previous study found that cisplatin-induced damage to the organ of Corti was primarily manifested by a loss of OHCs, with the severity of the loss progressively increasing from the apical to the basal turn (Breglio et al., 2017). Administration of sarsasapogenin significantly attenuated these effects and significantly improved the hearing threshold over a wide frequency range (8–30 kHz). In addition, sarsasapogenin prevented cisplatin-induced loss of both outer and inner hair cells, which is often associated with cisplatin-induced ototoxicity. These results suggested that sarsasapogenin not only protected the structural integrity of cochlear HCs but also preserved auditory function.

Accumulated evidence has demonstrated that oxidative stress, which leads to excessive accumulation of ROS, plays a crucial role in the pathogenesis of cisplatin-induced ototoxicity (Callejo et al., 2015; Gentilin et al., 2019; Rybak et al., 2019; Tan and Vlajkovic, 2023). The excessive production and accumulation of ROS can lead to the death of cochlear HCs through multiple mechanisms, including apoptosis, ferroptosis, and necroptosis (Wang et al., 2023; Zhang et al., 2024). Furthermore, excessive accumulation of ROS disrupts MMP, leading to mitochondrial dysfunction (Shan et al., 2022). Antioxidants are considered effective protective agents against CIHL (Kim et al., 2018). In this study, we observed a significant increase in ROS production after cisplatin treatment as shown by the DCFH-DA and Mito-SOX assays in HEI-OC1 cells. Importantly, sarsasapogenin treatment effectively suppressed ROS elevation. Mitochondria are considered the main source of ROS, and excessive accumulation of ROS leads to mitochondrial dysfunction (Fujimoto and Yamasoba, 2019). Our results also showed that mtROS levels were significantly increased and mitochondrial integrity was impaired after cisplatin exposure, as evidenced by mtROS overproduction and MMP depolarization. However, these deleterious effects were significantly attenuated by sarsasapogenin pretreatment, suggesting that it has the potential to protect mitochondrial function and mitigate ROS-induced damage. Taken together, these results demonstrated that sarsasapogenin effectively reduced oxidative stress and maintained mitochondrial function.

Apoptosis, a tightly regulated form of programmed cell death, plays a critical role in the pathogenesis of cisplatin-induced ototoxicity (Ruhl et al., 2019). This process is characterized by a series of tightly controlled molecular events that lead to cell death, which helps maintain tissue homeostasis under normal conditions. However, in the context of cisplatin-induced damage, excessive activation of apoptotic signaling pathways contributes to cochlear hair cell degeneration, ultimately leading to hearing loss (Wang et al., 2023). In our study, apoptosis in HEI-OC1 cells was assessed using FITC-Annexin V/PI staining and flow cytometry. Consistent with previous studies, our results revealed a significantly higher proportion of apoptotic cells in the cisplatin treatment group than in the control group (Li et al., 2024; Lu et al., 2024). In contrast, the proportion of apoptotic cells in the sarsasapogenin pretreated group was markedly lower than that in the cisplatin-only group, indicating that sarsasapogenin effectively inhibited cisplatin-induced apoptosis of HEI-OC1 cells. Moreover, sarsasapogenin significantly alleviated cisplatin-induced apoptosis in HEI-OC1 cells and cochlear explants *in vitro*, as evidenced by the levels of apoptosis-related genes, *PARP*, *Casp3, CYCS*, and *Bax*, and the anti-apoptotic gene *Bcl2*. Additionally, sarsasapogenin pretreatment significantly reversed the high expression levels of the apoptosis-related proteins, caspase3 and CYCS, which were induced by cisplatin both *in vitro* and *in vivo*. Collectively, these findings suggested that sarsasapogenin effectively attenuated cisplatin-induced ototoxicity by suppressing apoptosis.

Recent research has highlighted the involvement of ferroptosis in cisplatin-induced ototoxicity (Liu et al., 2024; Mei et al., 2020). Ferroptosis, a particular form of regulated, non-apoptotic cell death, can be triggered by small molecules such as cisplatin or by impairment of glutathione biosynthesis or GPX4 activity. This process leads to an iron-dependent increase in lipid ROS and the degradation of polyunsaturated fatty acids within the plasma membrane (Jiang et al., 2021). In particular, cisplatin was observed to induce ferroptosis in HEI-OC1 cells, as evidenced by increased lipid peroxidation, iron accumulation and a decrease in MMP (Jiang et al., 2021). In this model, cisplatin rapidly degrades endogenous glutathione, either through direct binding or through oxidative reactions, promoting iron (Fe^2+^) formation and increased lipid peroxidation–hallmarks of ferroptosis (Zhang et al., 2024). Our results showed that sarsasapogenin effectively attenuated the cisplatin-induced increase in Fe^2+^ levels in both HEI-OC1 cells and cochlear explants, which are key indicators of ferroptosis onset. Furthermore, sarsasapogenin treatment significantly upregulated the mRNA expression of *Gpx4*, a central gene responsible for controlling ferroptosis by neutralizing lipid peroxidation (Xue et al., 2023). WB analysis further confirmed these results and showed that sarsasapogenin restored GPX4 protein levels, which were significantly downregulated in cisplatin-treated HEI-OC1 cells, cochlear explants and mouse cochleae. The upregulation of GPX4, a crucial enzyme that reduces lipid hydroperoxides, suggested that sarsasapogenin could counteract the oxidative damage and cell death mechanisms triggered by ferroptosis. This protective role against cisplatin-induced ototoxicity highlighted the therapeutic potential of sarsasapogenin in preventing hearing loss by inhibiting cisplatin-induced ferroptosis, thereby preserving the integrity and function of cochlear HCs both *in vitro* and *in vivo*.

Systemic administration of cisplatin is commonly used to establish animal models of ototoxicity (Rosati et al., 2021), but this method has significant drawbacks. The presence of the blood-labyrinth barrier limits drug delivery to the inner ear, while systemic toxicity may cause damage to other organs, potentially confounding auditory-specific outcomes. Moreover, systemic exposure often leads to severe side effects such as weight loss and motor impairment (Fernandez et al., 2019), raising ethical concerns and increasing animal mortality before meaningful results can be obtained (DeBacker et al., 2020). To address these issues, we adopted a transtympanic administration strategy, which has been proven effective in inducing consistent cochlear damage without systemic toxicity (Jiang et al., 2023. This approach not only improves model reproducibility and animal survival but also aligns with the “3R” (Replacement, Reduction, and Refinement) principles, thus improving the ethical quality of the study (Nacher-Soler et al., 2021). Currently, there is increasing recognition that phytochemicals can alleviate the side effects of chemotherapeutic agents, including cisplatin-induced ototoxicity (Oyenihi and Smith, 2019). Compounds such as astaxanthin and polydatin have been shown to be able to reduce ototoxicity, primarily due to their potent antioxidant properties (Li et al., 2022; Nan et al., 2022). Sarsasapogenin which also possesses antioxidant effects, has emerged as a potential candidate for otoprotection. Clinically, it has been shown that transtympanic injection has proven to be an effective strategy for delivering otoprotective agents against cisplatin-induced ototoxicity. This method can achieve higher drug concentrations in the inner ear while maintaining the antitumor efficacy of cisplatin (He et al., 2009). Therefore, transtympanic delivery of sarsasapogenin is a viable option for future clinical application. Previous studies have demonstrated the protective effects of sarsasapogenin against diabetic retinopathy and ulcerative colitis when administered orally or intraperitoneally to mice, without reporting side effects (Choi, 2023; Mandlik et al., 2021). Furthermore, our results suggest that sarsasapogenin does not directly affect antitumor activity of cisplatin, positioning it as a preferred option for clinical trials to reduce cisplatin-induced ototoxicity. However, a limitation of this study is the lack of pharmacokinetic data on the distribution of sarsasapogenin in the cochlea after transtympanic injection. In addition, it would be interesting to visualize cisplatin after treatment with sarsasapogenin to see whether sarsasapogenin blocks cisplatin entry into cells or not. Future research should fill these gaps to validate its otoprotective potential.

## 5 Conclusion

**FIGURE 9:**
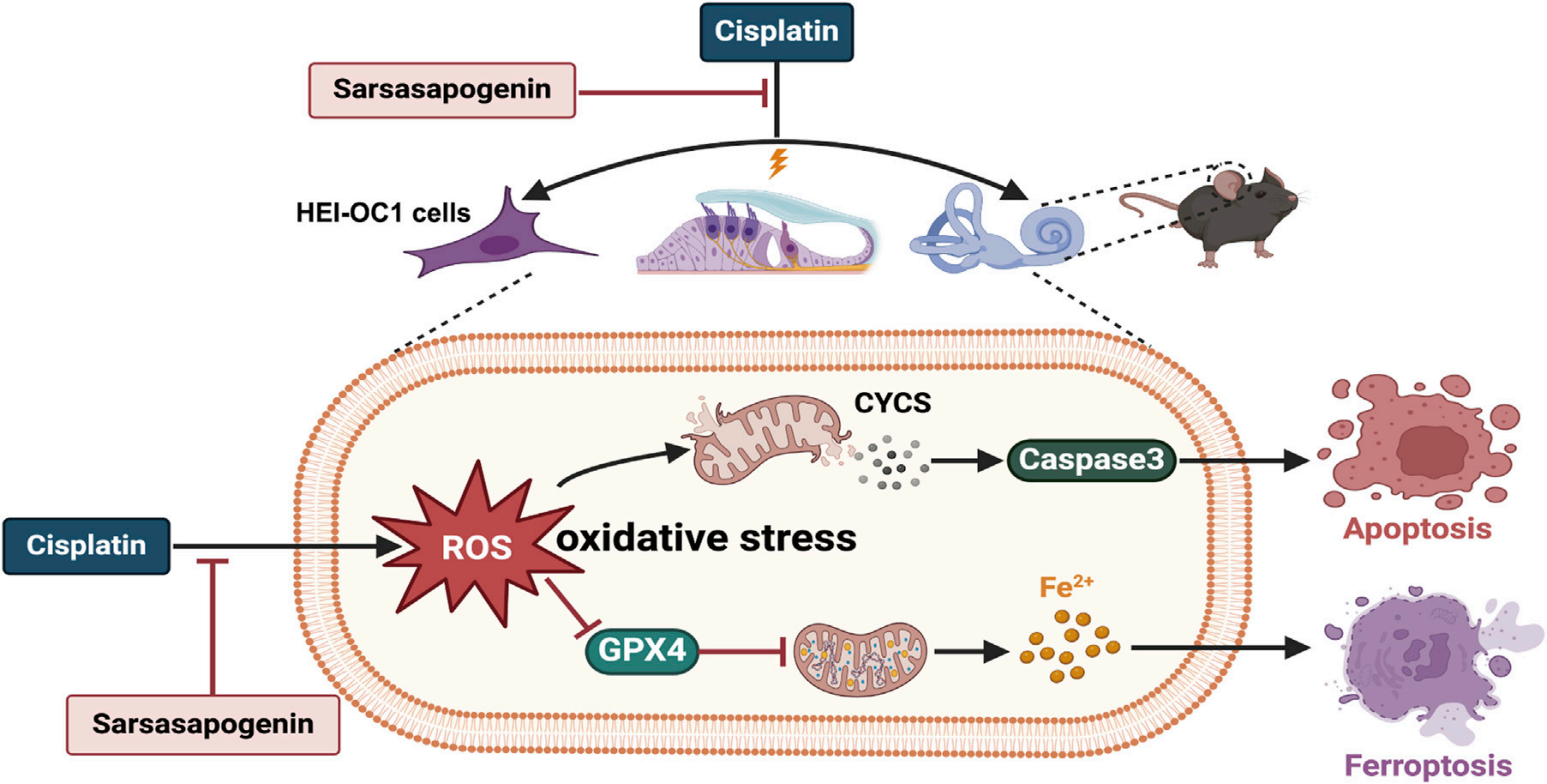
The schematic diagram of the possible mechanisms of the protective effect of sarsasapogenin on cisplatin-induced ototoxicity. Cisplatin increased the accumulation of ROS and Fe^2+^ as well as the expression levels of CYCS and caspase3, and decreased the expression level of GPX4 in cochlear hair cells, leading to oxidative stress and resulting in apoptosis and ferroptosis both *in vitro* and *in vivo*. Sarsasapogenin exerted a protective effect on cisplatin-induced ototoxicity by attenuating oxidative stress, apoptosis, and ferroptosis.

In summary, our study demonstrated for the first time that sarsasapogenin ameliorated cisplatin-induced ototoxicity by attenuating oxidative stress and preventing mitochondrial dysfunction. Mechanistically, the protective function of sarsasapogenin is attributed to the inhibition of apoptosis and ferroptosis both *in vitro* and *in vivo* (Figure 9). Furthermore, transtympanic delivery of sarsasapogenin in animal models demonstrated its potential to prevent cisplatin-induced ototoxicity, warranting further investigation in future studies. This study suggests that sarsasapogenin may serve as a promising therapeutic option for the prevention of cisplatin-induced ototoxicity.

## Data Availability

The raw data supporting the conclusions of this article will be made available by the authors, without undue reservation.
